# Impact of 1h oral glucose tolerance test on the clinical status of adult cystic fibrosis patients over a 4-year period

**DOI:** 10.1371/journal.pone.0246897

**Published:** 2021-03-18

**Authors:** Valérie Boudreau, Quitterie Reynaud, Angélique Denis, Johann Colomba, Sandrine Touzet, Katherine Desjardins, Stéphanie Poupon Bourdy, Isabelle Durieu, Rémi Rabasa-Lhoret

**Affiliations:** 1 Montreal Clinical Research Institute, Québec, Canada; 2 Département de nutrition et de Médecine, Université de Montréal, Montréal, Québec, Canada; 3 Centre de référence Adulte de la Mucoviscidose, Service de médecine interne, Hospices civils de Lyon, F-69495 Pierre Bénite, France; 4 Université de Lyon, Équipe d’Accueil Health Services and Performance Research (HESPER) 7425, Lyon, France; 5 Pôle de Santé Publique, Hospices Civils de Lyon, Lyon, France; 6 Équipe d’Accueil Health Services and Performance Research (HESPER) 7425, Université de Lyon, Lyon, France; 7 Cystic fibrosis clinic, Centre Hospitalier de l’Université de Montréal, Montréal, Québec, Canada; Laurentian University, CANADA

## Abstract

**Objective:**

To report the clinical profile associated with G60 and I60 over a 4-year prospective observational period in 2 large cohorts of adult patients with CF.

**Methods:**

319 patients were included (210 Canadian and 119 French) and classified according to their inclusion G60 (≥ or < 11.1 mmol/L) and the median inclusion I60 (≥ or < 24 mU/I). Forced expiratory volume in 1 second (FEV1), body mass index (BMI) were collected on OGTT days. Linear mixed regression models were used to assess the effect of G60 and I60.

**Results:**

High G60 was not associated to a lower FEV1 at inclusion and the follow-up decline was not higher in the high G60 group (Coefficient [95% CI]: -3.4 [-7.4;0.6], p = 0.0995.). There was no significant association between BMI and G60. Patients with high I60 tended to have a higher mean BMI (+0.5 kg/m^2^ [0.0 to 1.1], p = 0.05) but no interaction over time was observed.

**Conclusions:**

High G60 is not associated with a lower lung function at inclusion nor its decline over a 4-year follow-up. High I60 is slightly associated to a higher weight at inclusion, but not with BMI evolution over time in adult patients.

## Introduction

Cystic fibrosis (CF) is a genetic inherited disorder affecting approximatively one in 3800 people in the Caucasian population [1, 2] and leading to multiple organ damages with major damages to the lung and digestive systems [3]. CF transmembrane conductance regulator (CFTR) dysfunction also plays a role in the exocrine and endocrine pancreas insufficiency resulting, in combination with other factors including inflammation and oxidative stress, in a progressive decrease in insulin secretion [4]. Therefore, 20% of young adult CF patients have cystic fibrosis-related diabetes (CFRD) while the prevalence increases to almost 50% of people in their 50s. In addition, a similar proportion of patients have a glucose intolerance [5]. Hyperglycemia occurrence is associated with accelerated lung function decline as well as weight loss, thereby increasing early mortality [6, 7]. This association is at least partly explained by reduced insulin secretion leading to a reduced anabolic capacity [8].

Because of its frequency and possible impact on weight and lung function, it is recommended to screen annually for CFRD from the age of 10 years with a 2-hour oral glucose tolerance test (OGTT) [9]. The current OGTT glucose thresholds used for CFRD diagnosis are based on fasting and 2-h glucose values [10], but these are controversial because they are based on retinopathy risk in patients with type 2 diabetes. In CF, hyperglycemia is mainly associated with altered nutritional and pulmonary status [11]. Many efforts are being made to find if CF-specific glycemic thresholds could help to target patients at higher risk of clinical decline (nutritional and/or pulmonary status) before or at the time of CFRD onset. Indeed, many research groups have found that hyperglycemia at earlier OGTT time points (30, 60 or even 90 minutes) correlates better with CF clinical status decline than the standard 2-h diagnosis value. Interestingly, because most CF patients present a specific glucose excursion pattern characterized by normal fasting glucose followed by abrupt glucose excursion followed by a rapid decrease, a lot of these patients with frankly high glucose at intermediate time points are classified as having a normal glucose tolerance. In cross-sectional studies, these CF patients with a high 1h-OGTT glucose value (G60) or low plasma insulin value (I60) present reduced pulmonary function and/or weight, as observed in patients with *de novo* CFRD diagnosis [12–15]. One longitudinal study, however, showed no relationship between 1-h glucose values and pulmonary function evolution over a three-year follow-up period [16]. Prospective studies on large cohorts are still needed to confirm the clinical relevance of early time OGTT values to predict clinical decline over time.

The objective of this study is to describe the course of BMI and lung function evolution associated with hyperglycemia and hypoinsulinemia at 60 minutes of an OGTT test over a 4-year period in one large Canadian cohort and one large French cohort of adult patients with CF.

## Methods

### Study popualtion: GLYCONE database

Data from patients were obtained from two large prospective cohorts of CF patients (Montreal, Canada 2004–2016 and Rhône-Alpes region, France 2009–2012) previously described [17]. The corresponding institutional research ethics committee approved the protocol for each cohort and informed written consent has been obtained from all subjects included. The institutional review board of each participating hospital and research ethics board authorized the cohorts in accordance with the current ethical standards (Comité de Protection des Personnes in France, Comité d’éthique de la recherche in Canada), as well as the French data Protection Agency for DIAMUCO Cohort (Comission Nationale de l’Informatique et des Libertés CNIL). Additional administrative and ethical authorizations were obtained to create a common harmonized and secured database. All research was performed in accordance with relevant guidelines.

Briefly, main inclusion criteria were: patients over 18 of age with confirmed CF diagnosis, pancreatic insufficient with pancreatic enzyme supplementation, without known diabetes at inclusion, and clinically stable for at least one month before the OGTT visit. Main exclusion criteria were: previous diagnosis of diabetes, pregnancy, CF exacerbations in the past month or conditions that could interfere with glucose metabolism such as intravenous antibiotics, steroids (oral or intravenous), or growth hormone treatment. Patients diagnosed with confirmed *de novo* CFRD diagnosis during follow-up were excluded from further protocol visits and referred to an endocrinologist. The combined database included 371 patients; 42 were excluded due to a lack of FEV1, G60, and/or I60 data during follow-up, leaving 329 available patients: 210 Canadian patients and 119 French patients for the present analysis. The follow-up period was four years as the maximum follow-up of the French patients was 4 years.

### OGTT

Patients in both cohorts performed an annual OGTT (between 12 and 18 months between visits depending on clinical status). When not clinically stable (e.g. pulmonary infections, intravenous antibiotic, etc.), OGTT testing was postponed after 2 months of clinical stability. After an 8-hour fast, patients consumed a glucose beverage (1.75g/kg of body weight up to a maximum of 75g, 300 mL) in less than 5 minutes. Plasma glucose (glucose oxydase) and insulin (centralized dosage in Québec by BI-INS-IRMA; Cisbio Bioassays, France) values were measured before OGTT and then again at 60 (G60 for glucose & I60 for insulin) and 120 minutes. OGTTs were then performed annually for at least 4 years.

### Clinical data

On the day of the OGTT, patients performed a lung function test to assess pulmonary function by spirometry using the forced expiratory volume in 1 second in L (FEV1) and Hankinson 1999 formula for FEV1(%) [18]. Weight and height were measured. BMI was calculated using weight in kilograms divided by height in square meter (kg/m^2^). These values of FEV1 and BMI were then obtained annually on the day of OGTT testing. Bacterial colonization with *P*. *aeruginosa* and *S*. *aureus* in the year preceding the OGTT was also collected from medical files as well as data relating to hospitalization and intravenous antibiotic treatment.

### Statistical analysis

Descriptive statistics were used to summarize characteristics of patients at the year of entry into the cohort. Continuous data were presented as means and standard deviations; categorical data were presented as frequencies and percentage.

A linear mixed regression model with random intercept and random slope was fitted to assess the effect of G60 and I60 at inclusion in the cohort on the mean FEV1 at baseline and on the mean slope of FEV1 change over time. Baseline G60 and I60 were considered as categorical variables based on widely used threshold for G60 (≥ or < 11.1 mmol/L) (10) and median value for I60 (≥ or < 24 mU/L). Effects of G60 and I60 were controlled for the cohort, age, sex, Pseudomonas aeruginosa colonization, year of inclusion into the cohort (as medical care might have changed), and BMI at inclusion. Interaction between covariates (cohort, G60, and I60) and time were tested to characterize differences in longitudinal rates of change. After checking for linearity assumption, continuous variables were mean-centered (25 years old for inclusion age and 21 kg/m^2^ for BMI at inclusion). Nonsignificant covariates (p>0.05) were removed by backward elimination.

A similar approach was used to assess the association between G60 and I60 at inclusion in the cohort and BMI change over time. Interaction between covariates cohort, time, and gender were tested to control for difference between males and females. Age at inclusion was included as a categorical variable (<20 yrs, 20 to 29 yrs, 30 to 34 yrs, and ≥35 yrs) to account for the nonlinear relationship between BMI and age.

Results were interpreted with a 5% threshold for statistical significance. Statistical analyses were performed with SAS version 9.4 software (SAS Institute Inc.).

## Results

### Inclusion

A total of 329 patients were included: 210 Canadian CF patients and 119 French patients. Patients’ characteristics are described in Table 1. Mean age of participants at inclusion in the cohorts was 24.7 ± 6.3, 141 (43%) were women and 183 (56%) were F508del homozygous. At inclusion, mean FEV1% is 68.7 ± 19.9 and mean BMI was 21.0 ± 2.6 kg/m2. 213 (70%) of patients had chronic colonisation with pseudomonas at inclusion in the cohort. Mean plasma glucose (G60) at inclusion was 10.9 ± 3.0. 138 (42%) patients presented a G60 value above ≥ 11.1 mmol/L. Conversely, mean insulin (I60) at inclusion was 29.2 ± 19.3 mU/L. 160 patients (49%) had a value above the median (≥ 24mU/L).

**Table 1:** Patient characteristics at entry in each cohort.

	Total	Canada	France
Number of patients	329	210	119
Number of observations	978	572	413
Women, n(%)	141 (43)	90 (43)	51 (43)
Mean age (SD), yrs	24.7 (6.3)	24.5 (6.0)	24.9 (6.8)
F508del homozygous, n (%)	183 (56)	121 (58)	62 (52)
Colonization with *P*. *Aeruginosa*, n (%)	213 (70)	152 (72)	79 (66)
Colonization with *S*. *Aureus*, n (%)	199 (61)	115 (55)	84 (71)
FEV1, %, mean (SD)	68.7 (19.9)	71.4 (18.9)	63.9 (20.8)
BMI, kg/m^2^, mean (SD)	21.0 (2.6)	21.4 (2.8)	20.3 (2.2)
Courses of IV antibiotic per year, mean (SD)	0.7 (1.1)	0.6 (1.1)	0.8 (1.2)
PG60 ≥ 11.1 mmol/l, n (%)	138 (42)	104 (50)	34 (29)
IG60 ≥ 24 mU/l^3^, n (%)	160 (49)	112 (64°	48 (40)

Abbreviations: SD: Standard Deviation, FEV1: Forced expiratory volume in 1 second, PG60: OGTT 1-h glycemia value, IG60: OGTT 1-h insulinemia value, BMI: Body Mass Index.

### Glycemia at 60 minutes (G60) and pulmonary function over time

**Fig 1:**
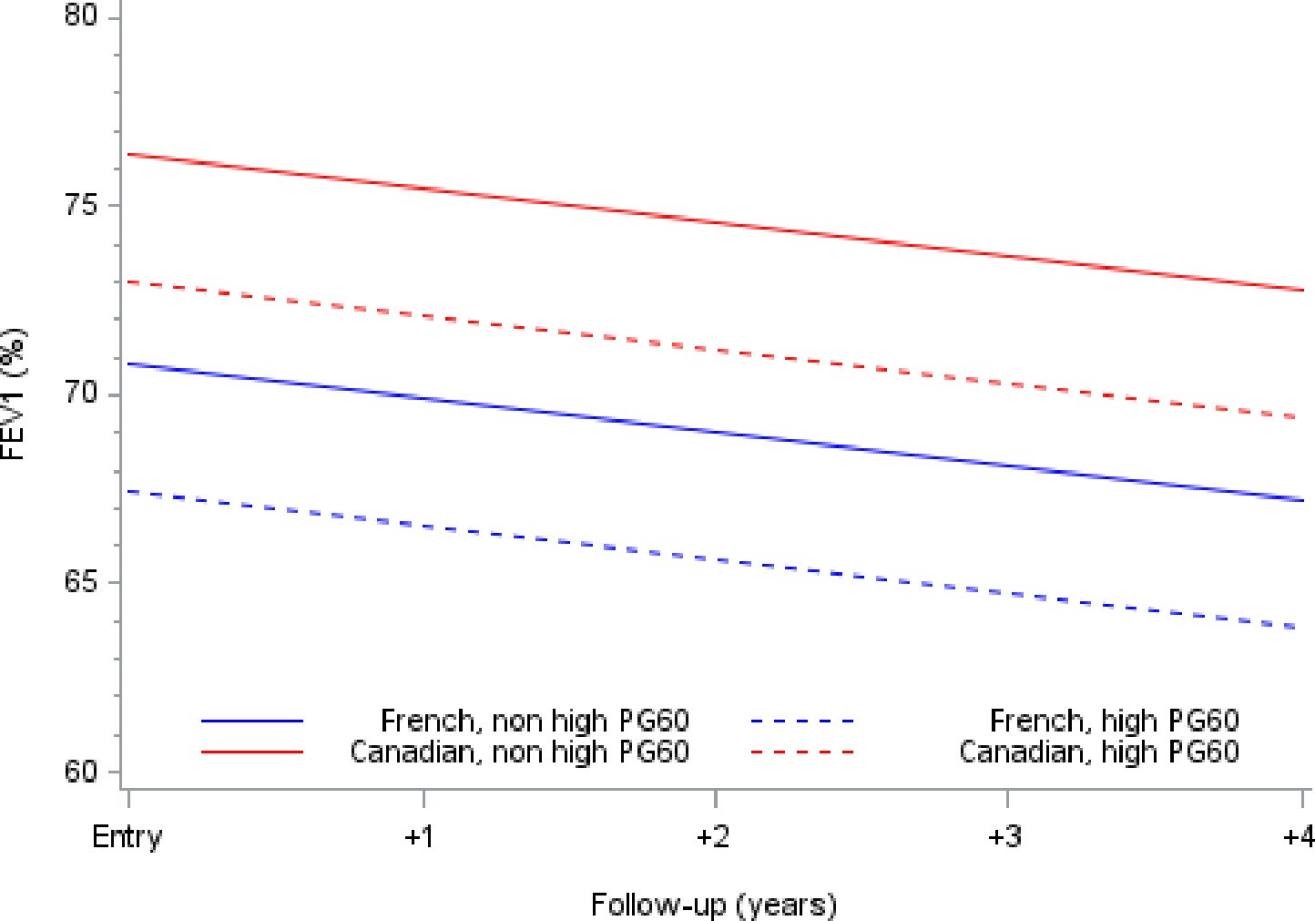
Prototypical FEV1 trajectories for Canadian and French patients according high PG60 values (≥11.1 mmol/l) at entry in each cohort.

FEV1 measurements over a 4-year follow-up period were obtained in the 329 patients, with a mean of 2.9 observations for each subject (range: 1–7), for a total of 981 observations. The overall mean decrease in FEV1 was 0.9% per year (95% CI: -1.3 to -0.4; p < 10^−3^). As previously reported, mean FEV1 at inclusion was significantly higher in Canadian patients than in French patients (+5.6%, 95% CI: [1.4; 9.8]; p < 0.001) (17). Patients with G60 values above 11.0 mmol/L tended to have a lower mean inclusion FEV1 than patients with G60 values below this threshold (-3.4%; 95% CI: -7.4 to 0.6; p<0.10). A similar non-significant trend was observed in patients with I60 values above the median (≥ 24mU/L) when compared with patients below this threshold (-2.8%; 95% CI: -6.8 to 1.3; p = 0.179). The relationship between the FEV1 decline and baseline G60 for Canadian and French patients is represented graphically in Fig 1. No significant interaction involving baseline G60 and FEV1 values over time was observed regardless of the cohort, indicating that the longitudinal changes in FEV1 over follow-up were not different between cohorts and not influenced by the baseline G60. The only factors significantly influencing mean FEV1 were age and BMI at baseline. Details are given in Table 2.

**Table 2:** Effect of PG60 at baseline on the change in FEV1 over time.

Factor	Coefficient [95% CI]	p-value
**PG60 on FEV1**		
Intercept^a^	70.8 [65.9;75.7]	< .0001
Time (year)	-0.9 [-1.3;-0.4]	0.0001
Canadian	5.6 [1.4;9.8]	0.0098
Baseline PG60 ≥11.1 mmol/l	-3.4 [-7.4;0.6]	0.0995
Baseline IG60 ≥24 mU/l^3^	-2.8 [-6.8;1.3]	0.1792
Baseline age (years) ^b^	-1.1 [-1.5;-0.8]	< .0001
Baseline BMI (kg/m^2^) ^c^	2.8 [2.0;3.6]	< .0001
Colonisation with *P*. *Aeruginosa*	-4.1 [-8.5;0.2]	0.0643
**IG60 on BMI**		
Intercept^d^	20.4 [19.5;21.3]	< .0001
Time (years)	0.2 [0.1;0.3]	< .0001
Canadian	1.7 [0.9;2.4]	< .0001
Women	-0.2 [-1.0;0.7]	0.0040
Women*Canadian	-1.3 [-2.3;-0.2]	0.0182
Time*Women	-0.1 [-0.3;0.0]	0.0446
Colonization with *P*. *Aeruginosa*	-0.7 [-1.3;-0.1]	0.0147
Age group		
20–29 yrs	-0.4 [-1.0;0.3]	0.3009
30–34 yrs	1.9 [0.9;2.9]	0.0002
≥35 yrs	1.5 [0.5;2.5]	0.0029
Baseline PG60 ≥11.1 mmol/l	-0.1 [-0.6;0.5]	0.7824
Baseline IG60 ≥24 mU/l^3^	0.5 [0.0;1.1]	0.0521

^a^ Mean FEV1 at entry in the cohort for a French patient aged 25 years, with BMI equals to 25 kg/m^2^, with no colonization with *P*. *Aeruginosa*, with a baseline PG60 < 11.1 mmol/l and with baseline IG60 < 24 mU/l^3^

^b^ Baseline age centered at 25 years

^c^ Baseline BMI centered at 21 kg/m^2^

^d^ Mean BMI at entry in the cohort for a French males aged 18 to 20 years with no colonization with *P*. *Aeruginosa*, with a baseline PG60 < 11.1 mmol/l and with baseline IG60 < 24 mU/l^3^

Abbreviations: CI: Confidence Interval, FEV1: Forced expiratory volume in 1 second, BMI: Body Mass Index, PG60: OGTT 1-h glycemia value, IG60: OGTT 1-h insulinemia value

### Insulin at 60 minutes (I60) and BMI over time

**Fig 2:**
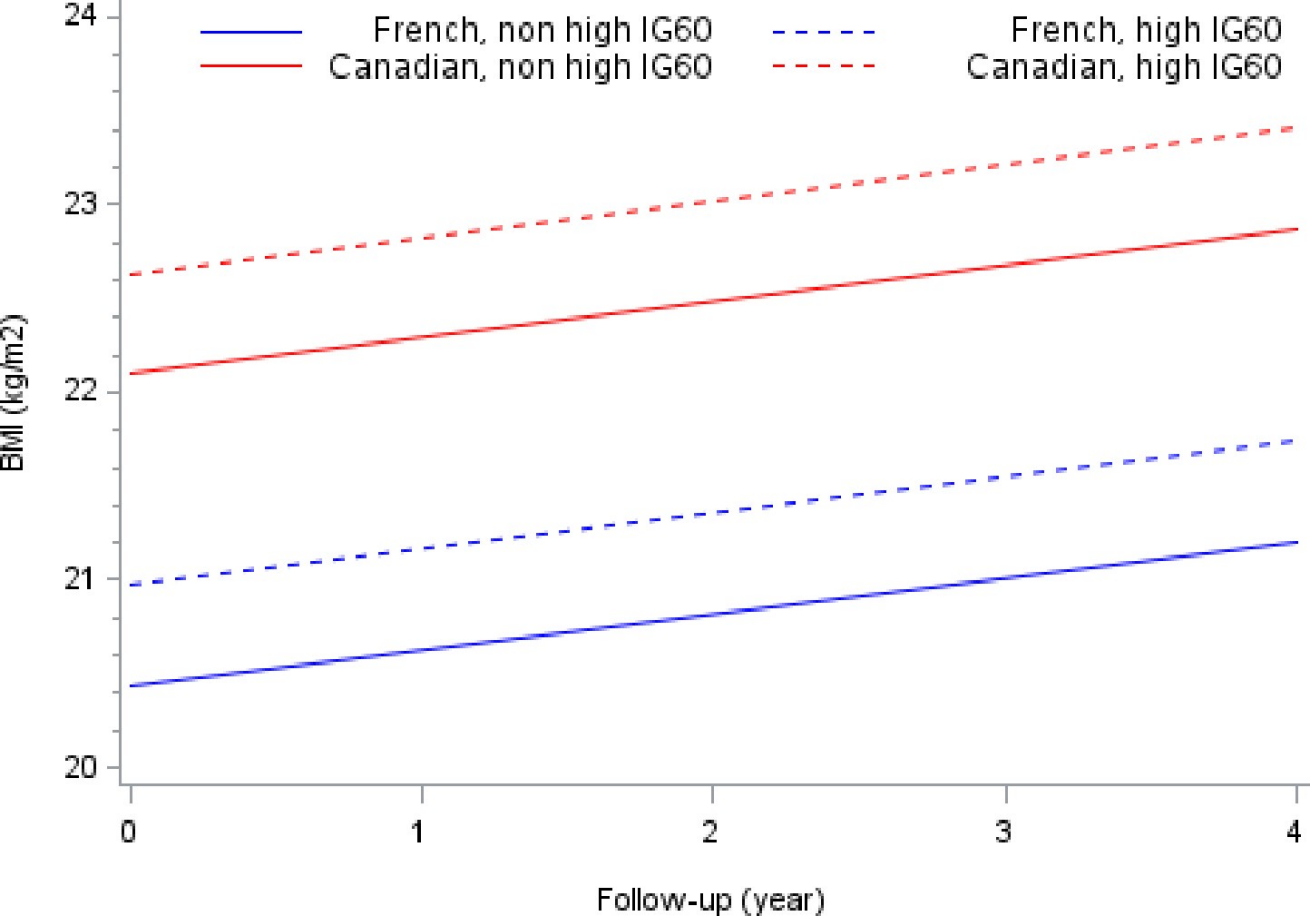
Prototypical BMI trajectories for Canadian and French patients according high IG60 values (≥ 24 mU/l^3^) at entry in each cohort.

BMI measurements over the 4-year follow-up period were obtained in 328 patients, with a mean of 2.9 observations for each subject (range: 1–7), for a total of 981 observations. As presented in Table 2, the linear mixed model depicted a significant gender difference in both the baseline mean BMI and the rate of change over the 4-year follow-up. Mean BMI was lower in females than males (- 0.2 kg/m^2^, 95% CI: 0.1 to 0.3; p < 10^−3^). The average rate of increase was + 0.2 kg/m^2^/year for males (95% CI: 0.1 to 0.3; p < 10^−3^) and + 0.1 kg/m^2^/year for females (95% CI: 0.0 to 0.2, p = 0.203). A cohort difference was found (p <10^−3^) and the effect was different between gender (p = 0.018). Canadian male patients had a significant higher mean BMI compared to French male patients (+1.7 kg/m^2^, 95% CI: 0.9 o 2.4, p<10^−3^). For females, the mean BMI was not different between Canadian and French patients (+0.4 kg/m^2^, 95% CI: -0.4 to 1.2, p = 0.328). There was no statistically significant association between BMI and G60 values at baseline above the threshold (p = 0.782). Patients with I60 values above 24mU/L tended to have a higher mean BMI than patients below this threshold (+0.5 kg/m^2^; 95% CI: 0.0 to 1.1, p = 0.05). In both cohorts, no interaction between time and I60 was observed, indicating that the rate of change of BMI was similar in patients above and below the I60 threshold. The relationship between the BMI change and baseline I60 for Canadian and French patients is represented graphically in Fig 2.

## Discussion

Our study did not support an association between baseline hyperglycemia at one hour with lung function decline neither in a French nor in a Canadian cohort. Low insulinemia at baseline tended to be associated with lower BMI at baseline but was not associated with decline of BMI over time. Despite a significant number of reports for significant associations between 1h-OGTT high glucose and/or low insulin values with lower pulmonary function and/or lower BMI [13–15], such relationship had not been tested prospectively. Only one longitudinal study showed there was no link between 1-h glucose values evolution over time and FEV1 evolution in adult patients over three year [16]. We used two large international CF cohorts to test longitudinally this hypothesis. We observed that 1) 1-h OGTT glucose values (G60) did not appear to influence FEV1% at cohort inclusion and during follow-up and 2) 1-h OGTT insulin values (I60) tended to significantly influence BMI at inclusion but not for evolution over time. As previously described with the same database [17], these data showed that Canadian patients, of a similar age and similar genotype, had better weight and lung function at inclusion than French patients at inclusion. However, regardless of G60, lung function decreased similarly over 4 years and weight increased in a similar way irrespectively of the French or Canadian origin.

The current 2h glucose-OGTT threshold (≥ 11.1 mmol/L) to diagnose CFRD was primarily established for type 2 diabetes diagnosis based on retinopathy risk [19]. Several groups have hypothesized that in patients with CF, the potential impact of hyperglycemia on lung function could occur at lower values, implying that current diagnosis may be made too late at a time at which the possible negative impact of hyperglycemia might be difficult to reverse [20, 21]. Though most studies are reporting fasting and 2h OGTT glucose values, the current mandatory values to establish glucose tolerance diagnosis, a lot of attention has been recently given to OGTT glucose intermediate times. In non-CF patients, intermediate points and especially 1-h glucose values, have been associated strongly and independently of 2-h glucose values to a wide variety of adverse outcomes [22]. Patients with CF present a specific post-meal or post-challenge glucose excursions characterized by frequent normal fasting glucose followed by early abrupt hyperglycemia and then rapid glucose value normalization [23, 24]; indeed, a specific glucose category exists for these patients (Indeterminate glucose tolerance; INDET) [12, 13]. Most studies showing that lower weight and lung function is associated with high G60 have been cross-sectional or sometimes retrospective [13–15]. Follow-ups over time following clinical parameters and 1-h glycemia are rare [15]. Several CGM studies exploring patients with CF have found that G60 was a better marker of clinical deterioration than the classical 2-h value [11]. Hameed et al. showed that the maximal blood glucose observed during continuous glucose monitoring (CGM) or OGTT was associated with worse weight and pulmonary function in children and adolescents, while the 2-h OGTT value was not [13]. Others have also shown that the INDET status is associated with reduced lung function and weight, to a comparable level to what is observed in CFRD patients [12]. The INDET status is a good illustration of some patients who can still normalize their 2-h glucose value but with early significant hyperglycemia which could affect clinical status [11, 20].

However, the current study does not agree with previous observations and does not confirm the deleterious effect of 1-h OGTT hyperglycemia (G60≥11.1 mmol/L) in adults with CF. At least in adult patients without known CFRD, this study may lead to reconsider the usefulness of G60 to detect the risk of future FEV1 decline. Still, at baseline a trend exists between a higher G60 value and a lower FEV1. It is thus possible that at a younger age before adulthood, hyperglycemia could have a more significant impact. This hypothesis was not confirmed in the study by Reynaud et al. [16], which included children with a three-year follow-up. Although we found that lung function may be lower at baseline when G60 is high, over a 4-year period the decline is not faster than for patients with normal G60, and weight may even increase over time. Since these patients are all adults, which is different from most of the previously published studies, it is possible that the consequences of hyperglycemia are mainly important during childhood and adolescence but then have less impact in adults.

In patients with CF, lower insulin values are associated and proportional to lower weight [14, 25]. This can be explained by the anabolic role of insulin [26]. The progressive loss of insulin secretion can lead to muscle and fat mass loss [5, 6]. Importantly, this process can be reversed by insulin therapy [7, 27]. However, regardless of the I60 values, patients included in this study gained weight over a 4-year period. One possible explanation is that since all patients are adults without CFRD at inclusion, these patients could still have sufficient insulin secretion to protect against protein catabolism [28]. It is possible that sicker patients in the low insulin secretion category developed CFRD before adult age and thus have not been included in this cohort.

Interestingly, both cohorts gained weight with time, a factor usually associated with better lung function [29, 30]. This weight gain was observed regardless the baseline weight and insulin secretion category. This positive weight trend could be related to intensive nutritional therapy in our cohorts with high caloric intake, enzyme replacement therapy, and nutritional follow-up in the context of reduced but sufficient insulin secretion. We observed a sex dysmorphism for weight gain which, over time, was more pronounced in men than in women. Lower weight has already been reported in women living with CF [6], but this might not be related to insulin secretion as in a previous report from the Canadian cohort, adult women surprisingly presented higher insulin secretion than adult men, and at a comparable level with what is observed in healthy individuals [31].

This study has several limitations. Inclusion criteria are strict since patients must be adults without CFRD at inclusion and without pulmonary transplantation. Thus, sicker patients diagnosed with CFRD or who underwent transplantation before adulthood are not included in this analysis. However, the investigated group represent a large proportion of adults followed in CF centers. Despite good phenotyping of our cohort, additional unmeasured parameters could influence FEV1 and BMI (e.g. nutritional intake, physical activity, modifier genes, etc.). Despite these limitations, both cohorts are well characterized with harmonized data allowing the prospective analysis of the impact of hyperglycemia and hypoinsulinemia on pulmonary function and weight evolution over time. Despite some baseline differences already reported^17^, overall results are similar in the two cohorts. This analysis does not identify high values of 1h glycemia and insulinemia as markers of clinical degradation and classical 2-hour OGTT measurements remain necessary to determine glycemic profiles of CF patients and its clinical impact. It also does not preclude a positive role for early insulin therapy on BMI and/or lung function. Glycemic profile of CF patients should be interpreted with caution, taking into account its evolution over time in association with clinical parameters.

In conclusion, hyperglycemia at one hour of an oral glucose tolerance test is not associated with a significant lower lung function at inclusion or decline over the subsequent 4 years, challenging its adverse effect on lung function in adult patients with CF. Low insulin value at one hour of an oral glucose tolerance test tends to be associated with lower BMI at baseline, but then patients increase their BMI over time regardless of their baseline insulin secretion.
